# Divergent risk profiles for cause-specific mortality in MASLD and MetALD: a nationwide population-based study

**DOI:** 10.3389/fmed.2026.1832416

**Published:** 2026-06-23

**Authors:** Hyuk Kim, Jeong-Ju Yoo, Young Chang, Jae Young Jang, Tom Ryu, Soung Won Jeong, Sang Gyune Kim, Young Seok Kim, Suyeon Park

**Affiliations:** 1 Division of Gastroenterology and Hepatology, Department of Internal Medicine, Soonchunhyang University Bucheon Hospital, Bucheon, Republic of Korea; 2 Department of Internal Medicine, Digestive Disease Center, Institute for Digestive Research, Soonchunhyang University College of Medicine, Seoul, Republic of Korea; 3 Department of Biostatistics, Soonchunhyang University Hospital, Seoul, Republic of Korea; 4 Department of Applied Statistics, Chung-Ang University, Seoul, Republic of Korea

**Keywords:** alcohol consumption, cardiometabolic risk factor, metabolic dysfunction-associated steatotic liver disease (MALSD), metabolic dysfunction-associated steatotic liver disease with increased alcohol intake (MetALD), mortality

## Abstract

**Background:**

Metabolic-dysfunction-associated steatotic liver disease (MASLD) and MASLD with increased alcohol intake (MetALD) are overlapping but distinct conditions that influence liver disease progression and mortality. This study sought to examine how differing metabolic, behavioral, and sociodemographic profiles influence mortality in MASLD and MetALD.

**Methods:**

Adults aged 20–79 years without a history of cancer, cardiovascular disease, or viral hepatitis were selected from the Korean National Health and Nutrition Examination Surveys (2007–2015). Mortality data up to November 2023 were obtained from the National Death Registry. MASLD and MetALD were defined using the hepatic steatosis index, cardiometabolic risk factors (CMRFs), and alcohol consumption.

**Results:**

A total of 7,783 participants, weighted to represent approximately 7.6 million Korean adults, were included, with a median follow-up duration of approximately 4 years. Both MASLD and MetALD were associated with increased all-cause mortality compared to controls, with a significant increase observed in MetALD (HR 2.085; 95% CI, 1.127–3.860; *p* = 0.019). The absolute all-cause mortality rates were 2.33% in the MetALD group and 1.05% in controls. Cancer-related mortality was significantly elevated in both MASLD (HR 2.610; 95% CI, 1.029–6.618; *p* = 0.043) and MetALD (HR 3.355; 95% CI, 1.097–10.263; *p* = 0.034). In MetALD, high alcohol consumption and current smoking were independent predictors of cancer-related and all-cause mortality. In MASLD, female sex and higher income were associated with reduced mortality. Cardiovascular mortality did not differ significantly between groups; however, in MASLD, each additional CMRF was associated with increased cardiovascular mortality (HR 1.838; 95% CI, 1.320–2.558; *p* < 0.001).

**Conclusion:**

While both MASLD and MetALD were associated with increased mortality, the contributing risk profiles differed. Alcohol-related behaviors predominated in MetALD, whereas metabolic and sociodemographic factors influenced outcomes in MASLD.

## Introduction

Metabolic-dysfunction-associated steatotic liver disease (MASLD), formerly known as non-alcoholic fatty liver disease, is a prevalent and clinically significant condition that affects approximately 27–30% of the population in South Korea (1–3). In 2023, the terminology was updated to MASLD to explicitly incorporate metabolic dysfunction into the disease definition. At the same time, the term metabolic-dysfunction-associated steatotic liver disease with increased alcohol intake (MetALD) was introduced to encompass individuals with both alcohol use and cardiometabolic risk factors (CMRFs) (4). Since this revision, research has focused primarily on the epidemiology of MASLD and its relationship with advanced fibrosis or hepatocellular carcinoma, while limited data exist on its impact on mortality. Moreover, available studies on mortality have yielded inconsistent findings. Some studies identified MASLD as a risk factor for both all-cause and cause-specific mortality (5, 6), while others linked increased mortality to MetALD, not MASLD (7). In addition, some studies implicate both conditions (8), and a meta-analysis identified MASLD as a risk factor for all-cause mortality (9). These discrepancies may partly reflect heterogeneity across studies, including differences in alcohol consumption cutoffs, definitions of cardiometabolic risk factors, and control group selection, which complicates the interpretation and clinical application of existing evidence.

The lack of standardized guidelines for managing mortality risks in patients with MASLD and MetALD underscores the urgent need for targeted research, particularly regarding the roles of CMRF and alcohol intake in determining long-term outcomes. Therefore, this study aimed to elucidate differences in cause-specific mortality—specifically cancer-related and cardiovascular mortality—as well as all-cause mortality in patients with MASLD and MetALD versus healthy controls. Additionally, it sought to examine the impact of CMRFs, alcohol intake, lifestyle and sociodemographic factors, including smoking status, income level, and educational attainment, on mortality using a large nationwide cohort.

## Materials and methods

### Study population

This study utilized data from the Korea National Health and Nutrition Examination Survey (KNHANES), an annual survey conducted by the Korea Disease Control and Prevention Agency to assess the health and nutritional status of the Korean population. Data from 2007 to 2015, encompassing KNHANES IV–VI, were included. Of 73,353 participants, 21,778 not registered in the National Death Registry were excluded. Mortality data were analyzed as of 12 December 2023.

**FIGURE 1:**
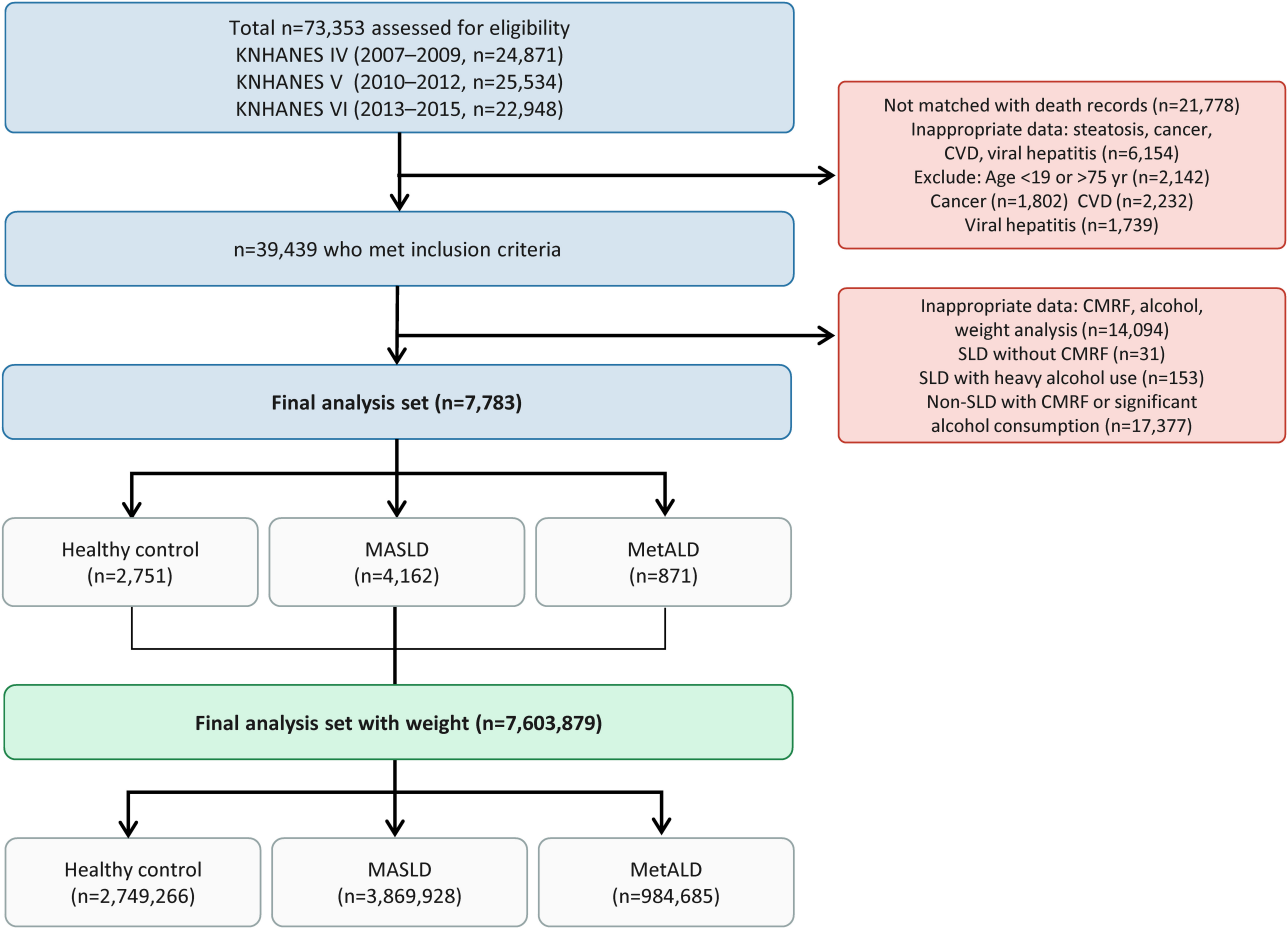
Flow diagram of patient inclusion. CVD, cardiovascular disease; SLD, steatotic liver disease; CMRF, cardiometabolic risk factor; MASLD, metabolic dysfunction-associated steatotic liver disease; MetALD, MASLD with increased alcohol intake.

Participants aged < 18 or > 74 years, those with insufficient data on liver or comorbid conditions, or a history of these conditions were excluded, as were those with incomplete data on CMRFs, alcohol use, or weighting variables. We also excluded those with SLD but no CMRFs or with heavy alcohol intake, and those with CMRFs or significant alcohol use but no SLD. The final cohort comprised 7,783 participants: 2,751 in the control group, 4,162 in the MASLD group, and 871 in the MetALD group. After weighting, this corresponded to an estimated 7,603,879 individuals: 2,749,266 controls, 3,869,928 with MASLD, and 984,685 with MetALD (Figure 1). The study was approved by the IRB of Soonchunhyang University Seoul Hospital (IRB No. SCHUH 2023-12-017, approved on December 29, 2023), in accordance with the Declaration of Helsinki. The requirement for written informed consent was waived by the IRB due to the retrospective nature of the study.

### Definitions

#### Steatotic liver disease and hepatic fibrosis

SLD was defined using the hepatic steatosis index (HSI), a well-validated non-invasive parameter for diagnosing fatty liver disease (10). The HSI was calculated using the following formula, with a diagnostic cut-off value set at >36:

HSI = 8 × [alanine transaminase (ALT)]/[aspartate transaminase (AST)] + [body mass index (BMI)] ( + 2 if diabetes patient, + 2 if female)

Hepatic fibrosis was assessed using the Fibrosis-4 (FIB-4) index, a widely accepted surrogate marker. The FIB-4 index was calculated as follows:


$$\mathrm{FIB}-4=\left(\mathrm{age}\times \mathrm{AST}\right)/\left(\mathrm{platelet}\,\mathrm{count}\times \sqrt{\mathrm{ALT}}\right)$$

A FIB-4 value >2.36 was considered indicative of advanced fibrosis.

#### CMRFs and alcohol consumption

CMRFs included BMI ≥ 23 kg/m^2^, abdominal obesity (WC > 90 cm for men, > 85 cm for women), impaired glucose metabolism (fasting glucose ≥ 100 mg/dL, HbA1c ≥ 5.7%, or diabetes), elevated blood pressure (≥ 130/85 mmHg or hypertension), triglycerides ≥ 150 mg/dL, and low HDL (≤ 40 mg/dL for men, ≤ 50 mg/dL for women).

Alcohol consumption was categorized by weekly consumption levels: non-significant (< 210 g for men, < 140 g for women), light to moderate (210–350 g for men, 140–280 g for women), high (350–420 g for men, 280–350 g for women), and heavy (> 420 g for men, > 350 g for women).

#### Group classification: control, MASLD, and MetALD

Participants were divided into three groups based on specific criteria. The control group comprised individuals with an HSI of ≤ 36, no CMRFs, and non-significant alcohol consumption. The MASLD group comprised those with an HSI of > 36, at least one CMRF, and non-significant alcohol intake. The MetALD group comprised participants with an HSI of > 36, at least one CMRF, and either light-to-moderate or high alcohol consumption.

### Study design and outcomes

The primary aim of this study was to compare mortality outcomes—specifically all-cause, cancer-related, and cardiovascular mortality—across MASLD, MetALD, and control groups. We also examined factors associated with each mortality outcome within the MASLD and MetALD populations. Risk factor analysis was conducted using a broad set of variables available in the KNHANES dataset, including sociodemographic characteristics (age, sex, education, income), behavioral factors (daily energy intake, alcohol consumption, smoking status), and a range of CMRFs.

### Statistical analysis

Due to the survey’s two-stage stratified sampling, composite sample analysis was conducted using integration weights (2007–2015) with a 1/9 integration ratio per year. Continuous variables are expressed as weighted means ± standard errors (SEs) and categorical variables as counts or percentages. Chi-square tests and analysis of variance were used for categorical and continuous variables, respectively. Both univariate and multivariate Cox regression analyses were performed to assess survival differences, with results displayed in Kaplan–Meier plots and expressed as odds ratios or hazard ratios (95% CIs). Subgroup analyses evaluated the impact of CMRFs and alcohol consumption on mortality outcomes. All analyses incorporated integrated survey weights for the complex sampling design. Statistical computations used IBM SPSS 25.0, R v3.6.1, and the R survey package (R v4.3.2), with statistical significance at *p* < 0.05.

## Results

### Baseline characteristics

Baseline characteristics are shown in Table 1. The mean age was 42.4 years in MASLD and 40.8 years in MetALD groups, both higher than controls (33.3 years). Male prevalence was highest in MetALD (85.2%), followed by MASLD (59.7%) and controls (36.7%). Both disease groups showed lower income and higher prevalence of glucose intolerance/diabetes (MASLD 42%, MetALD 50%), hypertension (MASLD 65%, MetALD 78%), and smoking (MetALD 63%). Biochemical markers including cholesterol, AST, and ALT were significantly elevated in MASLD and MetALD groups. Mean Fibrosis-4 index was higher in both disease groups than controls. Advanced fibrosis was rare in all groups (MASLD 0.48%, MetALD 0.47%, controls 0.30%).

**TABLE 1:** Baseline characteristics of the study population.

Variables	Control *n* = 2,749,266 (weighted)/2,751 (unweighted)	MASLD *n* = 3,869,928 (weighted)/4,162 (unweighted)	MetALD *n* = 984,685 (weighted)/871 (unweighted)	*p*-value
Age, years	33.3 ± 0.3	42.4 ± 0.3	40.8 ± 0.4	<0.001
Male	936,731 (34.1)	2,311,429 (59.7)	839,283 (85.2)	<0.001
Income level^a^				0.032
1st (lowest)	495,893 (18.3)	727,904 (19.0)	191,482 (19.6)	
2nd	498,058 (18.4)	835,665 (21.8)	206,767 (21.1)	
3rd	544,848 (20.1)	811,413 (21.2)	191,104 (19.5)	
4th	532,221 (19.6)	697,245 (18.2)	192,730 (19.7)	
5th (highest)	639,223 (23.6)	752,594 (19.7)	196,437 (20.1)	
Education level				<0.001
Elementary school	69,607 (2.5)	529,537 (13.7)	76,059 (7.7)	
Middle school	115,253 (4.2)	357,430 (9.2)	94,701 (9.6)	
High school	1,250,528 (45.6)	1,552,697 (40.2)	456,032 (46.5)	
College or higher	1,307,939 (47.7)	752,594 (36.9)	196,437 (36.1)	
Past medical history				
Diabetes or pre-diabetes	0 (0.0)	1,609,374 (41.6)	495,896 (50.4)	<0.001
Hypertension	91,612 (3.3)	2,488,184 (64.4)	763,348 (77.9)	<0.001
Hypercholesterolemia	55,846 (2.0)	678,519 (17.6)	171,604 (17.4)	<0.001
Cardiometabolic risk factors				
BMI, kg/m^2^	20.11 ± 0.04	27.83 ± 0.06	28.18 ± 0.13	<0.001
Waist circumference, cm	70.66 ± 0.15	91.34 ± 0.17	93.39 ± 0.33	<0.001
Fasting glucose, mg/dL	86.38 ± 0.16	103.92 ± 0.52	106.95 ± 1.03	<0.001
Systolic blood pressure, mmHg	103.76 ± 0.21	120.46 ± 0.28	124.61 ± 0.58	<0.001
Diastolic blood pressure, mmHg	68.18 ± 0.17	79.82 ± 0.21	84.68 ± 0.47	<0.001
Triglyceride, mg/dL	69.85 ± 0.69	171.81 ± 2.37	235.38 ± 7.51	<0.001
HDL-cholesterol, mg/dL	58.97 ± 0.24	43.97 ± 0.18	46.20 ± 0.43	<0.001
Alcohol consumption				<0.001
Light to moderate^b^	0 (0.0)	0 (0.0)	861,899 (87.5)	
High^c^	0 (0.0)	0 (0.0)	122,785 (12.5)	
Smoking				<0.001
Never-smoker	1,855,018 (67.5)	1,882,397 (48.7)	157,673 (16.0)	
Ex-smoker	224,217 (8.2)	529,583 (13.7)	202,763 (20.6)	
Current smoker	670,031 (24.4)	1,457,198 (37.7)	624,248 (63.4)	
Daily energy intake (kcal)	2011.10 ± 22.42	2087.13 ± 18.97	2522.96 ± 45.24	<0.001
Laboratory findings				
AST, IU/L	18.36 ± 0.16	26.07 ± 0.26	28.26 ± 0.65	<0.001
ALT, IU/L	14.43 ± 0.19	38.90 ± 0.58	41.00 ± 1.17	<0.001
Platelet count, × 10^9^/L	250.56 ± 1.16	265.11 ± 1.08	253.56 ± 2.02	<0.001
Fibrosis				
FIB-4 index	0.70 ± 0.01	0.75 ± 0.01	0.78 ± 0.02	<0.001
Advanced fibrosis^d^	7,174 (0.30)	16,579 (0.48)	4,137 (0.47)	0.604

Data are presented as weighted means ± standard errors for continuous variables and as numbers (percentages) for categorical variables.

*^a^*Income level was categorized into quintiles.

*^b^*Light to moderate alcohol consumption was defined as 210–350 g/week for males, and 140–280 g/week for females.

*^c^*High alcohol consumption was defined as 350–420 g/week for males, and 280–350 g/week for females.

*^d^*Advanced fibrosis was defined as an FIB-4 index of ≥ 2.67. MASLD, metabolic dysfunction-associated steatotic liver disease; MetALD, MASLD with increased alcohol intake; CMRF, cardiometabolic risk factor; BMI, body mass index; HDL, high-density lipoprotein; AST, aspartate transaminase; ALT, alanine transaminase; FIB-4 index, Fibrosis-4 index.

Factors associated with advanced fibrosis are summarized in Supplementary Table 1. In the overall population, older age and a higher number of CMRFs were significantly associated with advanced fibrosis. In the MASLD group, older age, female sex and a higher number of CMRFs were significantly associated with advanced fibrosis. In the MetALD group, older age remained significantly associated with advanced fibrosis, while a higher number of CMRFs and high alcohol consumption showed positive trends toward advanced fibrosis without statistical significance.

### Distribution of CMRFs

**FIGURE 2:**
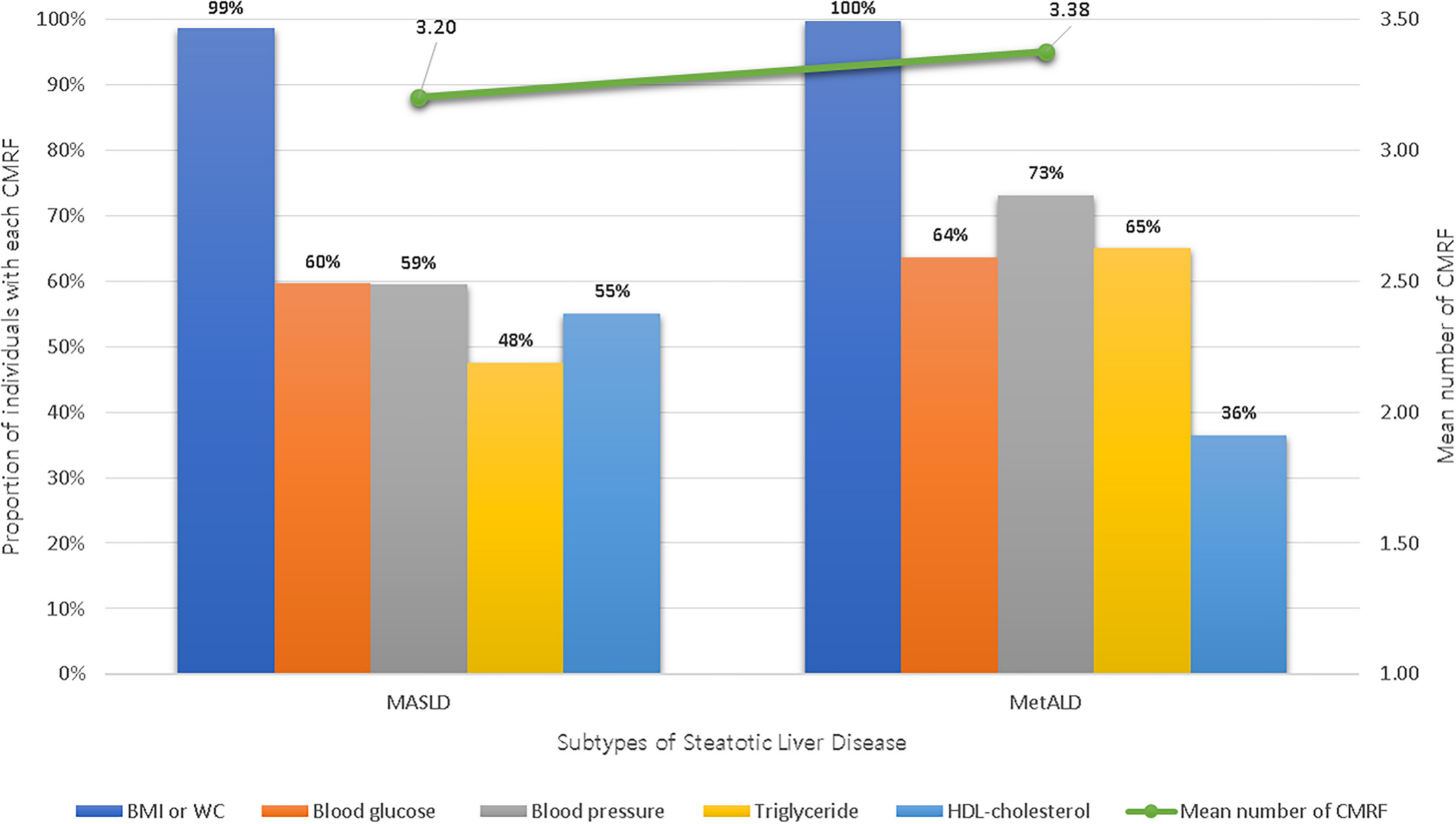
Distribution of cardiometabolic risk factors by types of steatotic liver disease. MASLD, metabolic-dysfunction associated steatotic liver disease; MetALD, MASLD with increased alcohol intake; BMI, body mass index; WC, waist circumference; HDL-cholesterol, high density lipoprotein-cholesterol; CMRF, cardiometabolic risk factor.

Figure 2 displays the distribution of CMRFs across the study groups. Elevated BMI or waist circumference was the most prevalent risk factor, with > 99% of patients in both the MASLD and MetALD groups meeting these criteria. Although risk factors were relatively evenly distributed across groups, some differences were observed in specific components. Patients in the MetALD group had a higher prevalence of elevated blood pressure (73.1% vs. 59.4%), triglycerides (64.9% vs. 47.5%), and a lower prevalence of low HDL (36.5% vs. 55.1%; all *p* < 0.001) compared to patients in the MASLD group. The difference in elevated blood glucose did not reach statistical significance (63.6% vs. 59.6%, *p* = 0.077). The mean number of CMRFs was higher in the MetALD group than in the MASLD group (3.38 vs. 3.20, *p* < 0.001).

### All-cause mortality

In the control group (*n* = 2,749,266), 28,812 (1.05%) patients died, with 17.86% of deaths attributed to cardiovascular causes and 20.67% to cancer. In the MASLD group (*n* = 3,869,928), 61,029 (1.58%) patients died. Among these, cardiovascular-related mortality accounted for 12.70%, while cancer-related mortality comprised 35.74% of deaths. In the MetALD group (*n* = 984,685), 22,960 (2.33%) patients died, with 31.36% of deaths attributed to cardiovascular causes and 30.91% to cancer-related causes (Supplementary Table 2). Corresponding incidence rates per 1,000 person-years were 2.58, 3.69, and 5.45 for all-cause mortality in the control, MASLD, and MetALD groups, respectively, over a mean follow-up duration of approximately 4 years. Cancer-related mortality rates were 0.75, 1.43, and 2.03, while cardiovascular mortality rates were 0.59, 0.53, and 2.10 per 1,000 person-years in the respective groups (Supplementary Table 3).

**FIGURE 3:**
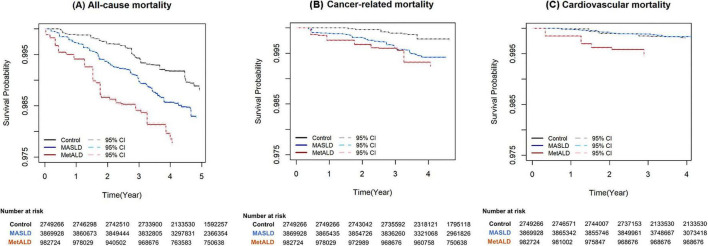
Kaplan–Meier plots for mortality by steatotic liver disease subgroup. **(A)** All-cause mortality. **(B)** Cancer-related mortality. **(C)** Cardiovascular mortality. MASLD, metabolic dysfunction-associated steatotic liver disease; MetALD, MASLD with increased alcohol intake.

Kaplan–Meier analysis showed a clear difference in all-cause mortality risk between groups (Figure 3A). The MetALD group had a significantly higher risk of all-cause mortality compared to controls (HR 2.085; 95% CI, 1.127–3.860; *p* = 0.019), whereas the MASLD group showed a non-significant trend toward increased risk (HR 1.527; 95% CI, 0.930–2.507; *p* = 0.094).

In MASLD, female sex was independently protective [adjusted HR (aHR) 0.190; 95% CI 0.086–0.419; *p* < 0.001], while lower socioeconomic status increased mortality risk (Table 2). Advanced fibrosis and elevated blood pressure were significant in univariate analysis but not independently predictive after adjustment. In MetALD, alcohol dose was not significantly associated with mortality, whereas current smoking remained a strong independent risk factor (aHR 17.130; 95% CI 2.169–135.306; *p* = 0.007; Table 3).

**TABLE 2:** Variables associated with all-cause mortality in MASLD.

Variables	Univariate analysis		Multivariate analysis^a^	
	HR (95% CI)	*p*-value	HR (95% CI)	*p*-value
Age, years	1.071 (1.050–1.092)	<0.001	1.081 (1.052–1.111)	<0.001
Female	0.608 (0.361–1.022)	0.061	0.190 (0.086–0.419)	<0.001
Income				
1st (lowest)	1 (ref)		1 (ref)	
2nd	0.478 (0.235–0.972)	0.042	0.367 (0.174–0.774)	0.009
3rd	0.550 (0.254–1.189)	0.128	0.340 (0.164–0.704)	0.004
4th	0.376 (0.182–0.777)	0.008	0.380 (0.169–0.852)	0.019
5th (highest)	0.815 (0.407–1.631	0.563	0.674 (0.305–1.488)	0.329
Education				
Elementary	1 (ref)			
Middle	0.629 (0.314–1.259)	0.191	0.639 (0.295–1.385)	0.257
High	0.338 (0.180–0.635)	0.001	0.575 (0.257–1.290)	0.180
College	0.296 (0.144–0.608)	0.001	0.463 (0.193–1.109)	0.084
Daily energy intake	0.787 (0.599–1.035)	0.087	1.001 (0.705–1.421)	0.997
Advanced fibrosis	7.239 (1.630–32.140)	0.009	1.651 (0.284–9.602)	0.577
Cardiometabolic risk factors				
Blood pressure	2.204 (1.166–4.164)	0.015	1.700 (0.858–3.367)	0.128
BMI or WC	0.578 (0.108–3.107)	0.523	1.722 (0.226–13.130)	0.600
Glucose	1.504 (0.866–2.610)	0.147	0.833 (0.451–1.540)	0.561
HDL	1.161 (0.703–1.919)	0.559	1.131 (0.678–1.886)	0.636
Triglyceride	0.757 (0.450–1.272)	0.293	0.717 (0.403–1.274)	0.257
Number of CMRFs	1.191 (0.956–1.483)	0.118		
Smoking				
Never smoker	1 (ref)		1 (ref)	
Ex-smoker	0.451 (0.178–1.142)	0.093	0.229 (0.076–0.690)	0.009
Current smoker	1.651 (0.986–2.766)	0.057	1.200 (0.517–2.787)	0.672

*^a^*Multivariate Model: Adjusted for age, sex, income, education, diet, fibrosis, CMRFs, and smoking. MASLD, metabolic dysfunction-associated steatotic liver disease; HR, hazard ratio; CI, confidence interval; BMI, body mass index; WC, waist circumference; HDL, high-density lipoprotein; CMRF, cardiometabolic risk factor; ref, reference.

**TABLE 3:** Variables associated with all-cause mortality in MetALD.

Variables	Univariate analysis		Multivariate model 1^a^		Multivariate model 2^b^	
	HR (95% CI)	*p*-value	HR (95% CI)	*p-*value	HR (95% CI)	*p-*value
Age, years	1.068 (1.021–1.118)	0.004	1.097 (1.055–1.142)	< 0.001	1.078 (1.030–1.127)	0.001
Female	–		–		–	
Income						
1st (lowest)	1 (ref)		1 (ref)			
2nd	2.598 (0.665–10.145)	0.170	0.718 (0.198–2.595)	0.613	0.881 (0.223–3.475)	0.856
3rd	2.708 (0.651–11.265)	0.171	0.287 (0.066–1.248)	0.096	0.361 (0.075–1.732)	0.203
4th	1.273 (0.246–6.587)	0.774	0.334 (0.079–1.416)	0.137	0.414 (0.107–1.601)	0.201
5th (highest)	1.006 (0.236–4.292)	0.993	0.159 (0.022–1.119)	0.065	0.221 (0.037–1.307)	0.096
Education						
Elementary	1 (ref)		1 (ref)			
Middle	0.254 (0.058–1.116)	0.070	0.460 (0.116–1.824)	0.269	0.491 (0.126–1.910)	0.305
High	0.293 (0.094–0.917)	0.035	0.630 (0.181–2.194)	0.468	0.617 (0.195–1.948)	0.410
College	0.246 (0.067–0.904)	0.035	1.269 (0.303–5.314)	0.745	1.361 (0.328–5.642)	0.671
Daily energy intake	0.904 (0.585–1.395)	0.647	1.069 (0.631–1.811)	0.804	1.080 (0.650–1.793)	0.767
Advanced fibrosis	4.564 (0.462–45.056)	0.194	0.562 (0.030–10.593)	0.700	0.362 (0.023–5.644)	0.468
Cardiometabolic risk factors						
Blood pressure	1.170 (0.387–3.535)	0.780	1.480 (0.361–6.073)	0.586		
BMI or WC	–		–			
Glucose	1.022 (0.380–2.749)	0.965	0.588 (0.187–1.850)	0.364		
HDL	0.721 (0.272–1.909)	0.510	0.747 (0.257–2.173)	0.592		
Triglyceride	0.644 (0.263–1.577)	0.336	0.395 (0.146–1.069)	0.067		
Number of CMRFs	0.885 (0.592–1.323)	0.550				
Alcohol consumption						
Light to moderate^c^	1 (ref)				1 (ref)	
High^d^	1.708 (0.535–5.457)	0.366			1.223 (0.278–5.387)	0.790
Smoking						
Never smoker	1 (ref)		1 (ref)		1 (ref)	
Ex-smoker	7.566 (0.849–67.440)	0.070	13.030 (1.085–156.503)	0.043	7.264 (0.787–67.023)	0.080
Current smoker	10.612 (1.385–81.289)	0.023	27.138 (2.742–268.631)	0.005	17.130 (2.169–135.306)	0.007

*^a^*Multivariate Model 1: Adjusted for age, sex, income, education, diet, fibrosis, CMRFs, and smoking.

*^b^*Multivariate Model 2: Adjusted for age, sex, income, education, diet, fibrosis, alcohol consumption, and smoking.

*^c^*Light to moderate alcohol consumption was defined as 210–350 g/week for males, and 140–280 g/week for females.

*^d^*High alcohol consumption was defined as 350–420 g/week for males, and 280–350 g/week for females. MetALD, metabolic dysfunction-associated steatotic liver disease with increased alcohol intake; HR, hazard ratio; CI, confidence interval; BMI, body mass index; WC, waist circumference; HDL, high-density lipoprotein; CMRF, cardiometabolic risk factor; ref; reference. “–” indicates that the statistic could not be reliably estimated because of the limited number of events.

### Cancer-related mortality

Cancer-related mortality was elevated in both the MASLD and MetALD groups compared to controls (Figure 3B). The MASLD group had a 2.6-fold increased risk (HR, 2.610; 95% CI, 1.029–6.618; *p* = 0.043), while the MetALD group had a 3.4-fold increase (HR, 3.355; 95% CI, 1.097–10.263; *p* = 0.034).

In MASLD, female sex was independently protective against cancer-related mortality (aHR 0.227; 95% CI 0.067–0.770; *p* = 0.017), as were higher income levels. College education showed univariate protection but did not remain significant after adjustment. Among CMRFs, elevated blood pressure and impaired glucose metabolism were associated with increased cancer mortality in univariate models but not in multivariate analysis. The overall number of CMRFs was not significantly associated with cancer-related mortality (Table 4). In MetALD, high alcohol consumption was a strong independent predictor of cancer-related mortality, conferring more than seven-fold increased risk compared to light-to-moderate consumption (aHR 7.837; 95% CI 2.169–28.320; *p* = 0.002; Table 5). CMRFs showed no association with cancer mortality in this group.

**TABLE 4:** Variables associated with cancer-related mortality in MASLD.

Variables	Univariate analysis		Multivariate analysis^a^	
	HR (95% CI)	*p-*value	HR (95% CI)	*p-*value
Age, years	1.119 (1.084–1.155)	<0.001	1.129 (1.089–1.170)	< 0.0001
Female	0.706 (0.334–1.495)	0.363	0.227 (0.067–0.770)	0.017
Income				
1st (lowest)	1 (ref)		1 (ref)	
2nd	0.254 (0.090–0.717)	0.010	0.165 (0.055–0.498)	0.001
3rd	0.314 (0.110–0.894)	0.030	0.137 (0.041–0.461)	0.001
4th	0.205 (0.063–0.666)	0.008	0.126 (0.029–0.550)	0.006
5th (highest)	0.293 (0.076–1.131)	0.075	0.205 (0.051–0.817)	0.025
Education				
Elementary	1 (ref)		1 (ref)	
Middle	0.634 (0.222–1.812)	0.395	1.023 (0.327–3.199)	0.969
High	0.252 (0.102–0.623)	0.003	1.085 (0.325–3.620)	0.895
College	0.137 (0.035–0.533)	0.004	0.856 (0.246–2.981)	0.807
Daily energy intake	0.905 (0.546–1.499)	0.698	1.433 (0.938–2.190)	0.097
Advanced fibrosis	5.599 (0.739–42.397)	0.095	0.565 (0.046–6.912)	0.655
Cardiometabolic risk factors				
Blood pressure	6.538 (1.532–27.902)	0.011	2.709 (0.649–11.310)	0.172
BMI or WC	0.235 (0.034–1.639)	0.144	–	
Glucose	2.583 (1.063–6.274)	0.036	0.724 (0.250–2.091)	0.550
HDL	0.771 (0.362–1.645)	0.502	0.992 (0.425–2.314)	0.985
Triglyceride	0.683 (0.315–1.483)	0.335	0.662 (0.295–1.483)	0.316
Number of CMRF	1.277 (0.921–1.771)	0.142		
Smoking				
Never smoker	1 (ref)		1 (ref)	
Ex-smoker	0.899 (0.277–2.924)	0.860	0.341 (0.071–1.639)	0.179
Current smoker	1.482 (0.659–3.331)	0.341	0.976 (0.211–4.505)	0.975

*^a^*Multivariate Model: Adjusted for age, sex, income, education, diet, fibrosis, CMRFs, and smoking. MASLD, metabolic dysfunction-associated steatotic liver disease; HR, hazard ratio; CI, confidence interval; BMI, body mass index; WC, waist circumference; HDL, high-density lipoprotein; CMRF, cardiometabolic risk factor; ref; reference. “–” indicates that the statistic could not be reliably estimated because of the limited number of events.

**TABLE 5:** Variables associated with cancer-related mortality in MetALD.

Variables	Univariate analysis		Multivariate model 1^a^		Multivariate model 2^b^	
	HR (95% CI)	*p-*value	HR (95% CI)	*p-*value	HR (95% CI)	*p-*value
Age, years	1.112 (1.044–1.184)	0.001	1.199 (1.116–1.289)	< 0.001	1.210 (1.129–1.298)	< 0.001
Female	–		–		–	
Income						
1st (lowest)	1 (ref)		1 (ref)		1 (ref)	
2nd	3.151 (0.529–18.782)	0.208	3.111 (0.547–17.705)	0.201	4.521 (0.784–26.060)	0.091
3rd	1.497 (0.216–10.382)	0.683	1.541 (0.157–15.116)	0.710	2.073 (0.204–21.094)	0.538
4th	0.180 (0.017–1.935)	0.157	0.118 (0.008–1.804)	0.124	0.099 (0.007–1.491)	0.095
5th (highest)	0.757 (0.071–8.035)	0.817	0.085 (0.002–4.050)	0.211	0.207 (0.004–10.256)	0.429
Education						
Elementary	1 (ref)		1 (ref)		1 (ref)	
Middle	0.287 (0.028–2.947)	0.294	0.212 (0.018–2.438)	0.213	0.448 (0.054–3.740)	0.458
High	0.171 (0.031–0.949)	0.043	0.755 (0.079–7.201)	0.807	0.880 (0.064–12.180)	0.924
College	0.335 (0.052–2.169)	0.251	5.065 (1.007–25.478)	0.049	10.861 (1.501–78.560)	0.018
Daily energy intake	0.637 (0.374–1.086)	0.097	0.523 (0.146–1.877)	0.320	0.639 (0.324–1.261)	0.197
Advanced fibrosis	–		–		–	
Cardiometabolic risk factors						
Blood pressure	3.618 (0.759–17.239)	0.106	2.547 (0.390–16.652)	0.329		
BMI or WC	–		–			
Glucose	0.880 (0.175–4.428)	0.877	0.264 (0.075–0.933)	0.039		
HDL	0.982 (0.201–4.797)	0.982	0.696 (0.227–2.137)	0.527		
Triglyceride	0.488 (0.113–2.104)	0.336	0.433 (0.115–1.634)	0.216		
Number of CMRFs	0.975 (0.549–1.734)	0.932				
Alcohol consumption						
*^c^*Light to moderate	1 (ref)				1 (ref)	
*^d^*High	7.632 (1.772–32.869)	0.006			7.837 (2.169–28.320)	0.002
Smoking						
Ex-smoker	1 (ref)		1 (ref)		1 (ref)	
Current smoker	2.424 (0.293–20.035)	0.411	8.154 (0.379–175.401)	0.180	9.548 (0.593–153.690)	0.111

*^a^*Multivariate Model 1: Adjusted for age, sex, income, education, diet, fibrosis, CMRFs, and smoking.

*^b^*Multivariate Model 2: Adjusted for age, sex, income, education, diet, fibrosis, alcohol consumption, and smoking.

*^c^*Light to moderate alcohol consumption was defined as 210–350 g/week for males, and 140–280 g/week for females.

*^d^*High alcohol consumption was defined as 350–420 g/week for males, and 280–350 g/week for females. MetALD, metabolic dysfunction-associated steatotic liver disease with increased alcohol intake; HR, hazard ratio; CI, confidence interval; BMI, body mass index; WC, waist circumference; HDL, high-density lipoprotein; CMRF, cardiometabolic risk factor; ref, reference. “–” indicates that the statistic could not be reliably estimated because of the limited number of events.

### Cardiovascular mortality

Regarding cardiovascular mortality, the MetALD group showed a non-significant trend toward increased risk compared to controls (HR, 2.901; 95% CI, 0.841–10.012; *p* = 0.092), while no difference was observed in the MASLD group (HR, 1.081; 95% CI, 0.399–2.929; *p* = 0.877; Figure 3C).

In the MASLD group, the number of CMRFs was significantly associated with cardiovascular mortality. Each additional CMRF increased the risk by 1.8-fold (HR, 1.838; 95% CI, 1.320–2.558; *p* < 0.001; Supplementary Table 4). Among individual CMRFs, elevated blood pressure was a significant predictor of cardiovascular mortality in both univariate and multivariate analyses (aHR, 5.566; 95% CI, 1.212–25.552; *p* = 0.027). Higher educational attainment was associated with reduced risk in univariate analysis, but income level showed no significant association. In contrast, in the MetALD group, neither the number of CMRFs nor alcohol consumption level was significantly associated with cardiovascular mortality (Supplementary Table 5).

### Stratified analyses by SLD subgroups

Stratified analyses of additional SLD subgroups excluded from the primary analysis are summarized in Supplementary Table 2. The SLD without CMRFs group (*n* = 51,497) had no deaths, preventing event rate estimation. In the SLD with significant alcohol consumption group (*n* = 180,805), 2,658 patients (1.47%) died. This group showed a higher hazard ratio for all-cause mortality compared with controls (HR 4.411; 95% CI 0.417–4.773; *p* = 0.580). Cardiovascular deaths comprised 35.78% of deaths in this group, with a higher hazard ratio for cardiovascular mortality (HR 2.818; 95% CI 0.329–24.154; *p* = 0.344), with no cancer-related deaths recorded. These elevated risks did not reach statistical significance, likely due to the relatively small sample size.

## Discussion

In this study, both the MASLD and MetALD groups exhibited higher mortality risks than did the control group, with distinct patterns across different causes of death. The MetALD group demonstrated a significant doubling of all-cause mortality, whereas there was a trend in the MASLD group toward a 50% increase, although this did not reach statistical significance. Cancer-related mortality was elevated in both groups, with a more than 2.6-fold increase in the MASLD group and a nearly 3.4-fold increase in the MetALD group. By contrast, neither MASLD nor MetALD was significantly associated with cardiovascular mortality. This may partly reflect the relatively young age of the study population, in whom long-term cardiovascular complications may not yet have fully manifested during the follow-up period.

In analyses of risk factors, female sex, higher income, and education were linked to lower all-cause and cancer-related mortality in MASLD, whereas current smoking and high alcohol intake were dominant predictors in MetALD. These findings suggest that in MASLD, sociodemographic factors are important modifiers of mortality risk, whereas in MetALD, lifestyle-related exposures such as smoking and alcohol intake may play more dominant roles. In MASLD, each additional CMRF was associated with a roughly 80% increase in cardiovascular mortality, with elevated blood pressure as a key independent factor. By contrast, no significant predictors of cardiovascular mortality were observed in MetALD. This may reflect distinct cardiovascular risk mechanisms in MetALD, potentially influenced by the complex effects of alcohol consumption on the cardiovascular system (11), as well as the predominance of male and high smoking prevalence in this group. Although female sex was associated with advanced fibrosis in MASLD, it was also associated with lower all-cause and cancer-related mortality. This discrepancy may suggest that female patients are more susceptible to hepatic fibrosis progression under metabolic stress, while favorable metabolic profiles and health-related behaviors could contribute to lower mortality risk (12, 13). Higher income was also associated with lower mortality, reflecting the potential impact of socioeconomic status on long-term outcomes through differences in healthcare access, health literacy, and lifestyle factors (14, 15).

Prior research on MASLD and MetALD has shown conflicting results, likely due to heterogeneity in study design, diagnostic criteria, and population characteristics. Some reported no increased mortality with MASLD (7), while others showed risk elevations in both groups (8). A previous study using nationwide database in Korea found MASLD, but not MetALD, to be a mortality risk factor—possibly due to differing inclusion criteria and definitions from our study (5). That study included participants of all ages including over 75 and did not control for CMRF or alcohol intake in the control group. More recent meta-analyses support MASLD as a risk factor for all-cause mortality, though substantial variability remains across studies (9).

CMRF burden has been shown to increase mortality in MASLD in previous studies (16–18). Our findings partially align, as this dose-dependent association was observed only for cardiovascular mortality (19). Consistent with this divergence, cancer-related mortality displayed a distinct pattern. A recent UK Biobank multi-cohort analysis reported elevated cancer mortality only in MASLD individuals with four or more CMRFs (20), consistent with our observation of a threshold rather than dose-dependent effect. While CMRFs may contribute beyond a certain level, cancer-specific factors likely play a more prominent role. Among CMRFs, elevated blood pressure and impaired glucose metabolism were particularly linked to adverse outcomes in MASLD, aligning with prior evidence (21, 22). Interestingly, although the overall burden of CMRFs was higher in the MetALD group than in the MASLD group, the number of CMRFs was not independently associated with mortality within the MetALD group. This may suggest that in MetALD, the prognostic impact of metabolic dysfunction is relatively attenuated by stronger competing lifestyle-related exposures, such as alcohol consumption and smoking, which were significant predictors of mortality in this group.

Regarding alcohol consumption, our findings differ from earlier studies suggesting no consistent dose-response effect (23). We observed proportional increases in mortality with greater alcohol consumption in MetALD, particularly for cancer-related mortality. Several studies have suggested a J-shaped association with lowest risk among light drinkers (24). However, such findings may be affected by misclassification bias, particularly abstainer bias. A recent meta-analysis addressing both misclassification and confounding demonstrated clear dose-dependent increases in cancer mortality with rising alcohol intake (25), supported by a large prospective cohort study (26). The relationship between alcohol and cardiovascular mortality is more complex. Proposed protective effects include improved lipid profiles and anti-inflammatory effects indicated by lower C-reactive protein and interleukin-6 (27, 28). However, these benefits are offset by alcohol’s direct toxic effects, including increased risks of atrial fibrillation, stroke, heart failure, and cardiomyopathy (27, 29). These competing effects explain the lack of clear association in our study. In contrast, the stronger cancer-related mortality association reflects alcohol’s carcinogenic properties in the context of metabolic dysfunction.

Smoking was also a major cancer-related mortality predictor in MetALD, with > 10-fold risk increases (30). While smoking has been linked to increased mortality in NAFLD, its specific impact on cause-specific mortality has been less clear (30, 31). The particularly strong association with cancer-related mortality in MetALD may reflect synergistic carcinogenic mechanisms. Tobacco smoke induces DNA damage and promotes oncogenesis, while high alcohol intake amplifies these effects through acetaldehyde accumulation and oxidative stress, enhancing mutagenesis and inflammation (32). This synergy likely accelerates hepatic carcinogenesis and smoking-related malignancies. While smoking trends for all-cause and cardiovascular mortality were less pronounced, this may reflect competing risks or baseline risk masking, consistent with prior studies showing smoking disproportionately affects cancer outcomes in metabolic liver disease (33).

Socioeconomic factors, particularly income level, were significant predictors of mortality in MASLD. In our analysis, higher income was independently associated with a lower risk of all-cause and cancer-related mortality in MASLD, while educational attainment showed a similar directional trend without statistical significance after multivariable adjustment. These findings are in line with previous studies demonstrating that lower income and education levels are linked to poorer clinical outcomes and reduced access to care in patients with MASLD (34, 35). These findings reinforce the importance of addressing not only metabolic but also structural determinants in MASLD management.

This study used KNHANES data, which employs nationally representative, stratified sampling to estimate health indicators across the general population. Unlike disease-specific registries or hospital-based cohorts, KNHANES provides insight into MASLD and MetALD impact in community-dwelling populations. While MASLD has been extensively studied, MetALD remains relatively underexplored in large-scale, population-based settings. This study adds valuable population-level insights on MetALD mortality risk using nationally representative data.

This study has several limitations. First, KNHANES lacks imaging or histological data for assessing SLD severity. We used the validated Hepatic Steatosis Index (10), widely applied in epidemiologic research (36, 37). However, some degree of misclassification may have occurred because HIS is an indirect surrogate marker. To improve diagnostic specificity, hepatic steatosis was defined using a high HSI cutoff in this study, and the control group was restricted to individuals without any CMRFs or significant alcohol consumption. Nevertheless, both false-negative and false-positive misclassification may still have occurred. Therefore, future imaging- or biopsy-based validation studies are warranted. Second, alcohol consumption and smoking status were self-reported, introducing potential recall or social desirability bias. In particular, under-reporting of alcohol intake may have resulted in misclassification of some individuals with higher alcohol consumption into lower intake categories, potentially leading to underestimation of the true impact of alcohol on mortality outcomes. Nevertheless, most large-scale epidemiological studies rely on self-reported data, and KNHANES minimizes such bias through standardized data collection methods with documented reliability. Finally, while KNHANES provides extensive health data, generalizability may be limited as it primarily represents a Korean population. Integrated sampling weights improved population-level representativeness, though applicability beyond Asian populations remains uncertain. Longer-term prospective studies are warranted to clarify these associations.

In conclusion, patients with MASLD and MetALD had higher all-cause and cancer-related mortality rates than healthy controls, with the risk being particularly pronounced in the MetALD group. In MASLD, prognosis was primarily influenced by the burden of CMRFs and sociodemographic factors, whereas in MetALD, alcohol consumption emerged as the key determinant of mortality. From a clinical perspective, these findings support distinct management priorities between MASLD and MetALD, with greater emphasis on metabolic risk reduction in MASLD and prioritization of alcohol-related risk modification in MetALD.

## Data Availability

The data analyzed in this study is subject to the following licenses/restrictions: The data used in this study are derived from the Korea National Health and Nutrition Examination Survey (KNHANES) linked with mortality data from Statistics Korea. Due to legal and privacy restrictions, the linked dataset is not publicly available. Access to the data can be obtained through the Korea Disease Control and Prevention Agency (KDCA) upon reasonable request and with appropriate approval from the relevant authorities. Requests to access these datasets should be directed to Young Chang, chyoung86@gmail.com.
