# Supplementary material for: Divergent risk profiles for cause-specific mortality in MASLD and MetALD: a nationwide population-based study
Source: Front Med (Lausanne). 2026 Jun 23;13:1832416. doi: 10.3389/fmed.2026.1832416 (PMC13337397; doi:10.3389/fmed.2026.1832416)
Supplement: Supplementary file 1 [file Data_Sheet_1.docx]

**Supplementary Table**

**Supplementary Table 1.** Factors associated with advanced fibrosis^a^ in MASLD and MetALD

|  | Overall | | MASLD | | MetALD | |
| --- | --- | --- | --- | --- | --- | --- |
|  | OR (95% CI) | *p-*value | OR (95% CI) | *p-*value | OR (95% CI) | *p-*value |
| Age, years | 1.125 (1.079–1.174) | <0.001 | 1.153 (1.106–1.202) | <0.001 | 1.152 (1.084–1.225) | <0.001 |
| Female | 1.384 (0.657–2.913) | 0.392 | 3.463 (1.391–8.622) | 0.008 | 1.025 (0.096–10.927) | 0.983 |
| Number of CMRF | 1.336 (1.026–1.783) | 0.031 | 1.792 (1.196–2.683) | 0.005 | 4.775 (0.877–26.002) | 0.071 |
| Alcohol consumption |  |  |  |  |  |  |
| Insignificant | 1 (ref) |  |  |  |  |  |
| Light to moderate^a^ | 0.616 (0.188–2.011) | 0.422 |  |  | 1 (ref) |  |
| High^b^ | 5.038 (0.674–37.652) | 0.115 |  |  | 8.183 (0.842–79.518) | 0.070 |
| Smoking |  |  |  |  |  |  |
| Never smoker | 1 (ref) |  | 1 (ref) |  | 1 (ref) |  |
| Ex-smoker | 0.316 (0.098–1.023) | 0.055 | 0.150 (0.019–1.161) | 0.069 | 0.153 (0.009–2.488) | 0.187 |
| Current smoker | 1.042 (0.483–2.246) | 0.916 | 0.783 (0.317–1.937) | 0.597 | 1.448 (0.132–15.918) | 0.762 |

^a^Advanced fibrosis was defined as an FIB-4 index of ≥ 2.67.

^b^Light to moderate alcohol consumption was defined as 210–350 g/week for males, and 140–280 g/week for females.

^c^High alcohol consumption was defined as 350–420 g/week for males, and 280–350 g/week for females.^c^

Abbreviations: MASLD, metabolic dysfunction-associated steatotic liver disease; MetALD, metabolic dysfunction-associated steatotic liver disease with increased alcohol intake; OR, odds ratio; CI, confidence interval; CMRF, cardiometabolic risk factor; ref, reference

**Supplementary Table 2.** Mortality according to steatotic liver disease status and cause of death

|  | **Control**  **(n = 2,749,266)** | | **MASLD**  **(n = 3,869,928)** | | **MetALD**  **(n = 984,685)** | | **SLD without CMRFs (n=51,497)** | | **SLD with significant alcohol consumption (n=180,805)** | |
| --- | --- | --- | --- | --- | --- | --- | --- | --- | --- | --- |
|  | n | % | n | % | n | % | n | % | n | % |
| **All-cause mortality** | 28,812 | 1.05 | 61,029 | 1.58 | 22,960 | 2.33 | 0 | 0 | 2,658 | 1.47 |
| **Cancer-related mortality** | 5,954 | 0.22 | 21,814 | 0.56 | 7,097 | 0.72 | 0 | 0 | 0 | 0 |
| **Proportion of mortality** |  | 20.67 |  | 35.74 |  | 30.91 |  |  |  |  |
| **Cardiovascular mortality** | 5,145 | 0.19 | 7,748 | 0.20 | 7,200 | 0.73 | 0 | 0 | 951 | 0.53 |
| **Proportion of mortality** |  | 17.86 |  | 12.70 |  | 31.36 |  |  |  | 35.78 |

Abbreviations: MASLD, metabolic dysfunction-associated steatotic liver disease; MetALD, MASLD with increased alcohol intake; SLD, stestotic liver disease; CMRF, cardiometabolic risk factor

**Supplementary Table 3.** Comparison of crude and incidence mortality rates with mean follow-up duration across steatotic liver disease subgroups

| Mortality Type | Subgroup | Crude mortality (%) | Person-years | Incidence rate (per 1,000 PY) | Mean follow-up duration (years) |
| --- | --- | --- | --- | --- | --- |
| All-cause | MASLD | 1.58 | 16,520,611.2 | 3.69 | 4.27 |
|  | MetALD | 2.33 | 3,855,915.0 | 5.45 | 3.92 |
|  | Control | 1.05 | 11,158,127.2 | 2.58 | 4.06 |
| Cancer-related | MASLD | 0.56 | 15,280,924.8 | 1.43 | 3.95 |
|  | MetALD | 0.72 | 3,500,822.9 | 2.03 | 3.57 |
|  | Control | 0.22 | 7,904,391.5 | 0.75 | 2.88 |
| Cardiovascular | MASLD | 0.20 | 14,495,139.9 | 0.53 | 3.75 |
|  | MetALD | 0.73 | 2,499,699.2 | 2.10 | 2.55 |
|  | Control | 0.19 | 8,680,955.7 | 0.59 | 3.16 |

Abbreviations: PY, person-years; MASLD, metabolic dysfunction-associated steatotic liver disease; MetALD, MASLD with increased alcohol intake

**Supplementary Table 4.** Variables associated with cardiovascular mortality in MASLD

|  | Univariate analysis | | Multivariate analysis^a^ | |
| --- | --- | --- | --- | --- |
|  | HR (95% CI) | *p-*value | HR (95% CI) | *p*-value |
| Age, years | 1.115 (1.060–1.173) | < 0.001 | 1.103 (1.033–1.177) | 0.003 |
| Female | 0.767 (0.302–1.949) | 0.578 | 0.300 (0.038–2.372) | 0.254 |
| Income |  |  |  |  |
| 1^st^ (lowest) | 1 (ref) |  | 1 (ref) |  |
| 2^nd^ | 0.258 (0.058–1.148) | 0.075 | 0.276 (0.064–1.187) | 0.084 |
| 3^rd^ | 0.403 (0.092–1.764) | 0.228 | 0.375 (0.085–1.661) | 0.196 |
| 4^th^ | 0.651 (0.141–3.011) | 0.582 | 0.593 (0.121–2.908) | 0.520 |
| 5^th^ (highest) | 0.702 (0.193–2.553) | 0.592 | 0.580 (0.146–2.305) | 0.439 |
| Education |  |  |  |  |
| Elementary | 1 (ref) |  | 1 (ref) |  |
| Middle | 0.228 (0.049–1.056) | 0.059 | 0.282 (0.036–2.231) | 0.230 |
| High | 0.193 (0.060–0.622) | 0.006 | 0.648 (0.097–4.303) | 0.653 |
| College | 0.105 (0.023–0.489) | 0.004 | 0.378 (0.055–2.578) | 0.320 |
| Daily energy intake | 0.581 (0.304–1.109) | 0.100 | 0.630 (0.237-–1.674) | 0.354 |
| Advanced fibrosis | – |  | – |  |
| Cardiometabolic risk factors | |  |  |  |
| Blood pressure | 11.722 (2.679–51.302) | 0.001 | 5.566 (1.212–25.552) | 0.027 |
| BMI or WC | – |  | – |  |
| Glucose | 2.544 (0.747–8.667) | 0.135 | 1.579 (0.326–7.663) | 0.570 |
| HDL | 2.107 (0.797–5.571) | 0.133 | 2.889 (0.979–8.521) | 0.055 |
| Triglyceride | 0.924 (0.343–2.485) | 0.875 | 0.623 (0.240–1.616) | 0.330 |
| Number of CMRFs | 1.838 (1.320–2.558) | < 0.001 |  |  |
| Smoking |  |  |  |  |
| Never smoker | 1 (ref) |  | 1 (ref) |  |
| Ex-smoker | 0.734 (0.091–5.948) | 0.772 | 0.442 (0.044–4.420) | 0.487 |
| Current smoker | 2.237 (0.848–5.901) | 0.104 | 2.877 (1.720–11.496) | 0.135 |

^a^Multivariate Model: Adjusted for age, sex, income, education, diet, fibrosis, CMRFs, and smoking.

Abbreviations: MASLD, metabolic dysfunction-associated steatotic liver disease; HR, hazard ratio; CI, confidence interval; BMI, body mass index; WC, waist circumference; HDL, high-density lipoprotein; CMRF, cardiometabolic risk factor; ref, reference

“–” indicates that the statistic could not be reliably estimated because of the limited number of events.

**Supplementary Table 5.** Variables associated with cardiovascular mortality in MetALD

|  | Univariate analysis | | Multivariate model 1^a^ | | Multivariate model 2^b^ | |
| --- | --- | --- | --- | --- | --- | --- |
|  | HR (95% CI) | *p-*value | HR (95% CI) | *p-*value | HR (95% CI) | *p-*value |
| Age, years | 1.109 (1.062–1.157) | < 0.001 | 1.135 (1.061–1.214) | <0.001 | 1.106 (1.049–1.166) | <0.001 |
| Female | – |  | – |  | – |  |
| Income |  |  |  |  |  |  |
| 1^st^ (lowest) | 1 (ref) |  | 1 (ref) |  | 1 (ref) |  |
| 2^nd^ | 0.086 (0.009–0.804) | 0.031 | 0.044 (0.002–1.043) | 0.053 | 0.090 (0.009–0.940) | 0.044 |
| 3^rd^ | – |  | – |  | – |  |
| 4^th^ | 0.179 (0.019–1.679) | 0.132 | 0.145 (0.004–5.475) | 0.297 | 0.319 (0.028–3.613) | 0.356 |
| 5^th^ (highest) | – |  | – |  | – |  |
| Education |  |  |  |  |  |  |
| Elementary | 1 (ref) |  | 1 (ref) |  | 1 (ref) |  |
| Middle | 0.119 (0.011–1.333) | 0.084 | 0.170 (0.013–2.224) | 0.177 | 0.178 (0.014–2.324) | 0.188 |
| High | 0.139 (0.022–0.886) | 0.037 | 0.679 (0.121–3.810) | 0.660 | 0.581 (0.084–4.019) | 0.582 |
| College | – |  | – |  | – |  |
| Daily energy intake | 0.593 (0.453–0.776) | <0.001 | 0.664 (0.402–1.098) | 0.111 | 0.712 (0.529–0.958) | 0.025 |
| Advanced fibrosis | – |  | – |  | – |  |
| Cardiometabolic risk factors | |  |  |  |  |  |
| Blood pressure | 1.325 (0.148–11.832) | 0.801 | 0.753 (0.103–5.483) | 0.779 |  |  |
| BMI or WC | – |  | – |  |  |  |
| Glucose | – |  | – |  | - |  |
| HDL | 1.288 (0.207–8.004) | 0.786 | 0.657 (0.090–4.819) | 0.680 |  |  |
| Triglyceride | 0.528 (0.091–3.066) | 0.477 | 0.166 (0.010–2.824) | 0.214 |  |  |
| Number of CMRF | 1.365 (0.580–3.216) | 0.476 |  |  |  |  |
| Alcohol consumption |  |  |  |  |  |  |
| Light to moderate^c^ | 1 (ref) |  |  |  | 1 (ref) |  |
| High^d^ | 0.587 (0.066–5.230) | 0.633 |  |  | 0.374 (0.037–3.745) | 0.403 |
| Smoking |  |  |  |  |  |  |
| Never smoker | 1 (ref) |  | 1 (ref) |  | 1 (ref) |  |
| Ex-smoker | 4.290 (0.387–47.497) | 0.235 | 21.171 (0.519–864.174) | 0.107 | 6.732 (0.625–72.475) | 0.116 |
| Current smoker | 1.655 (0.157–17.425) | 0.675 | 8.509 (0.435–166.238) | 0.158 | 3.372 (0.222–51.138) | 0.381 |

^a^Multivariate Model 1: Adjusted for age, sex, income, education, diet, fibrosis, CMRFs, and smoking.

^b^Multivariate Model 2: Adjusted for age, sex, income, education, diet, fibrosis, alcohol consumption, and smoking.

^c^Light to moderate alcohol consumption was defined as 210–350 g/week for males, and 140–280 g/week for females.

^e^High alcohol consumption was defined as 350–420 g/week for males, and 280–350 g/week for females.

Abbreviations: MetALD, metabolic dysfunction-associated steatotic liver disease with increased alcohol intake; HR, hazard ratio; CI, confidence interval; BMI, body mass index; WC, waist circumference; HDL, high-density lipoprotein; CMRF, cardiometabolic risk factor; ref, reference

“–” indicates that the statistic could not be reliably estimated because of the limited number of events.
